# Specificity Responses of Grasshoppers in Temperate Grasslands to Diel Asymmetric Warming

**DOI:** 10.1371/journal.pone.0041764

**Published:** 2012-07-27

**Authors:** Tingjuan Wu, Shuguang Hao, Osbert Jianxin Sun, Le Kang

**Affiliations:** 1 State Key Laboratory of Integrated Management of Insect Pests & Rodents, Institute of Zoology, Chinese Academy of Sciences, Beijing, China; 2 Institutes of Forestry & Climate Change Research, Beijing Forestry University, Beijing, China; Stanford University, United States of America

## Abstract

**Background:**

Global warming is characterized by not only an increase in the daily mean temperature, but also a diel asymmetric pattern. However, most of the current studies on climate change have only concerned with the mean values of the warming trend. Although many studies have been conducted concerning the responses of insects to climate change, studies that address the issue of diel asymmetric warming under field conditions are not found in the literature.

**Methodology/Principal Findings:**

We conducted a field climate manipulative experiment and investigated developmental and demographic responses to diel asymmetric warming in three grasshopper species (an early-season species *Dasyhippus barbipes*, a mid-season species *Oedaleus asiaticus*, and a late-season species *Chorthippus fallax*). It was found that warming generally advanced the development of eggs and nymphs, but had no apparent impacts on the hatching rate of eggs, the emergence rate of nymphs and the survival and fecundity of adults in all the three species. Nighttime warming was more effective in advancing egg development than the daytime warming. The emergence time of adults was differentially advanced by warming in the three species; it was advanced by 5.64 days in *C. fallax*, 3.55 days in *O. asiaticus*, and 1.96 days in *D. barbipes*. This phenological advancement was associated with increases in the effective GDDs accumulation.

**Conclusions/Significance:**

Results in this study indicate that the responses of the three grasshopper species to warming are influenced by several factors, including species traits, developmental stage, and the thermal sensitivity of the species. Moreover, species with diapausing eggs are less responsive to changes in temperature regimes, suggesting that development of diapausing eggs is a protective mechanism in early-season grasshopper for avoiding the risk of pre-winter hatching. Our results highlight the need to consider the complex relationships between climate change and specificity responses of invertebrates.

## Introduction

Global mean temperature has increased by 0.7°C since 1850 and is expected to rise 1.8–4.0°C by the end of this century [1]. Many studies and field experimental observation have indicated that global warming has significant effects on the structure and function of terrestrial ecosystems. Global warming is characterized by not only an increase in the daily mean temperature, but also a diel asymmetric pattern [2], [3], with greater trends of night warming than day warming [4]–[6]. The differential effects of asymmetric day and night warming on terrestrial ecosystem structure and functioning have been documented. Over the past decades, many studies concerning the impact of climate change were focused on vegetation and selected aboveground ecological processes [5]–[11]. However, it remains unclear whether asymmetrical warming may have different effects on other organism and belowground biological processes.

Enhancing downward infrared radiation from climate warming is responsible for increases in air temperature and soil microclimate [12]. Climate warming has been found to directly affect growth, metabolic and developmental processes in many plant, microorganism, and animal species. A diel asymmetric warming has been found to impose differential effects on plant production, soil microclimate, and soil CO_2_ emissions [2], [5], [7], [10], [13]. Recent studies show that day, night and continuous warming had different effects on longevity of plant roots and soil microbial composition in Inner Mongolian grasslands [14], [15]. Generally, daytime warming increases the maximum daily temperatures, thus exacerbates the daily temperature variation; whereas nighttime warming is associated with increases in the daily minimum temperatures, meaning a reduced day/night temperature fluctuation. For insect development, an increase in temperature near the lower temperature threshold can be more critical than at higher temperatures [16]. Differing from growing plants, which occur above- and belowground simultaneously during life cycle, grasshoppers spend half a year (i.e. from autumn to next spring) as eggs in soils and occur aboveground as hoppers and adults in growth seasons. As such, grasshoppers depend more on soil conditions than aboveground climate compared with plants. Therefore, an asymmetric day and night warming could have important implications to grasshoppers. Our previous study showed that constant warming significantly advanced embryonic development and hatching time but exerts little impact on nymphs and adults of grasshoppers [17]. To the best of our knowledge, a few studies have evaluated the effect of asymmetrically diel warming on eggs belowground and hoppers and adults aboveground.

In the temperate grasslands of Inner Mongolia, nearly all grasshopper species are univoltine and overwinter as eggs in soil [18]. The eggs of grasshoppers stay in soil for about half a year before hatching [19], and their hatching time is affected by temperature and water conditions [20], [21]. The grasshoppers occur in different season, form a sequential development, and have different egg diapauses traits (i.e. obligate, facultative, and non-diapause), with which grasshoppers determine their overwintering embryonic stage [18]. Our previous study showed that the late season grasshopper species was advanced more in phenology than early season grasshopper species by artificially constant warming [17]. The diapause trait was explained as the main reason for contrasting responses of grasshoppers to warming, because the developmental process was stopped and temperature accumulation was no means for embryonic development during diapause for early occurring grasshopper species [17]. Moreover, the responses of grasshopper eggs to warming differ from that of nymphs and adults due to their different habitat environment. Considering the habitat and seasonal climate variation, the different life history strategies of insect, and the differential effects of daytime and nighttime warming on soil and air temperature [2], it may be hypothesized that daytime warming and nighttime warming contribute differentially to developmental stage of the insects due to time of warming over a day [22], [23], and seasonal contrasting insect species would respond differentially to varying temperature regimes. Here we conduct a field climate manipulation experiment and investigate developmental and demographic responses to diel asymmetric warming in three grasshopper species with different life-history traits (*i.e.* an early-season species *Dasyhippus barbipes*, a mid-season species *Oedaleus asiaticus*, and a late-season species *Chorthippus fallax*) in temperate grassland of Inner Mongolia, northern China. We aim at determining: (1) whether the daytime and nighttime warming regimes would impose differential effects on the development, survival and reproduction of grasshoppers, (2) if diapause would limit the degree to which elevated temperature advances development, and (3) if there are species- and stage-specific responses to diel asymmetric warming in grasshoppers.

## Results

### Environmental impacts of warming treatments

**Figure 1:**
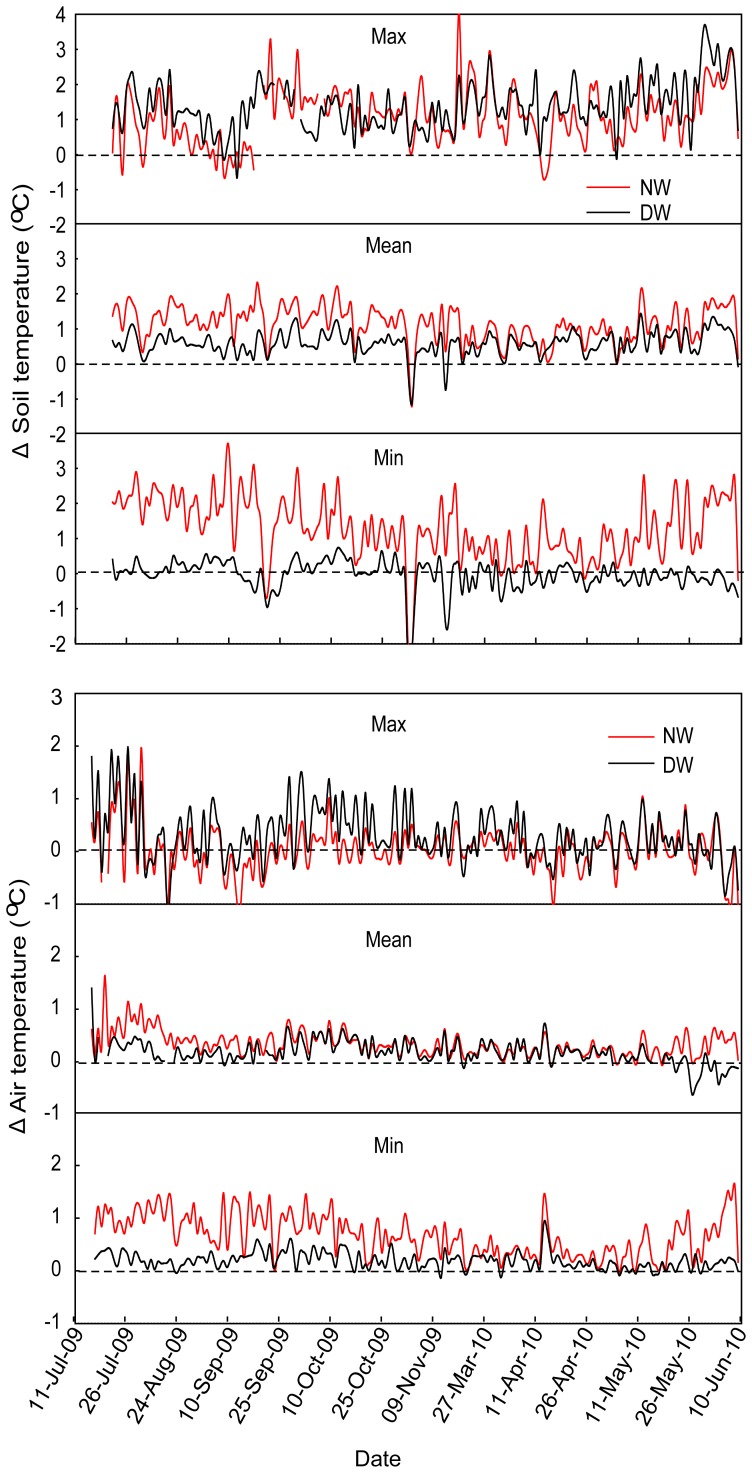
The variation of daily mean, maximum and minimum temperature of soil at 5 cm belowground and air at 10 cm aboveground. Δ (delta values) is calculated by subtracting average values in W0 from DW and NW treatments. Note: W0, control; DW, daytime warming; NW, nighttime warming;.

**Figure 2:**
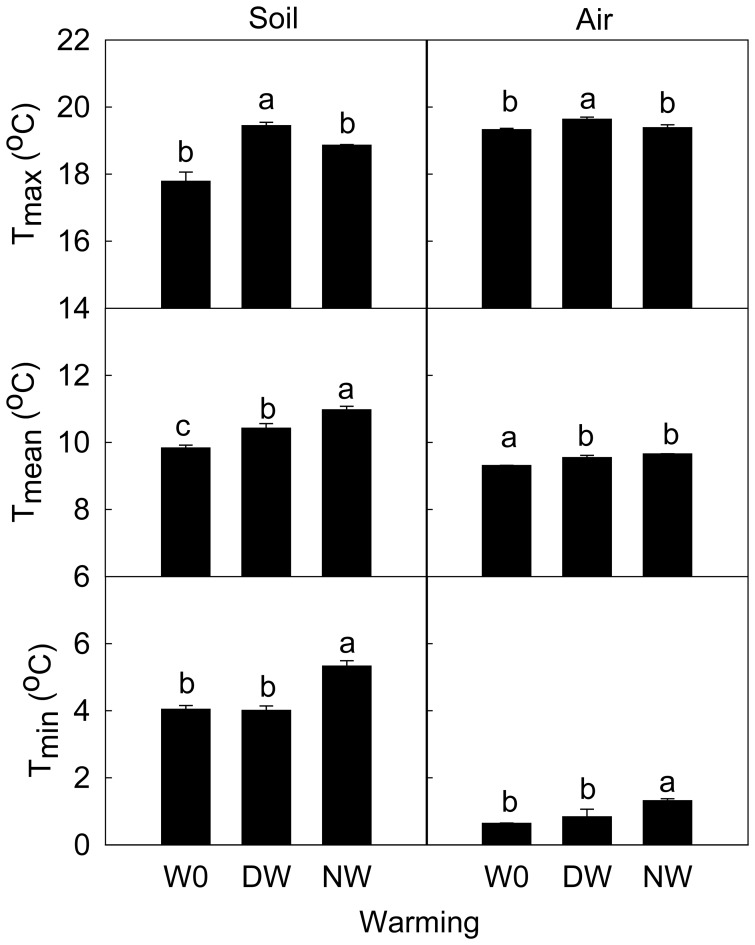
The variations of mean, maximum and minimum temperature of soil at 5 cm belowground and air at 10 cm aboveground in different warming treatments. Data are mean ± SE. Different letters indicate significantly different at *P*<0.05. Note: W0, control; DW, daytime warming; NW, nighttime warming.

The warming treatments effectively increased both soil and air temperatures during the field experimental period (Fig. 1 & 2). Daily mean soil temperature was significantly increased by 0.59°C by the daytime warming and 1.14°C by the nighttime warming (*F* = 27.02, *P* = 0.001). The maximum soil temperature was increased by 1.66°C by the daytime warming and 1.08°C by the nighttime warming (*F* = 18.77, *P* = 0.005). The minimum soil temperature was increased by 1.29°C by the nighttime warming ( = 29.29, *P* = 0.001), but not affected by the daytime warming (Fig. 2).

Over the experimental period, warming significantly increased the daily mean air temperature (*F* = 23.37, *P* = 0.001). The daily mean air temperature was increased by 0.23°C by daytime warming and 0.34°C by nighttime warming, but no significant difference was detected between the two warming regimes. The daily maximum air temperature was increased by 0.31°C (*F* = 5.85, *P* = 0.039) by the daytime warming, but not affected by the nighttime warming; whereas the minimum air temperature was significantly increased by 0.68°C (*F* = 6.50, *P* = 0.032,) by the nighttime warming, but not affected by the daytime warming (Fig. 1 & 2).

### Egg development and hatching rate

**Figure 3:**
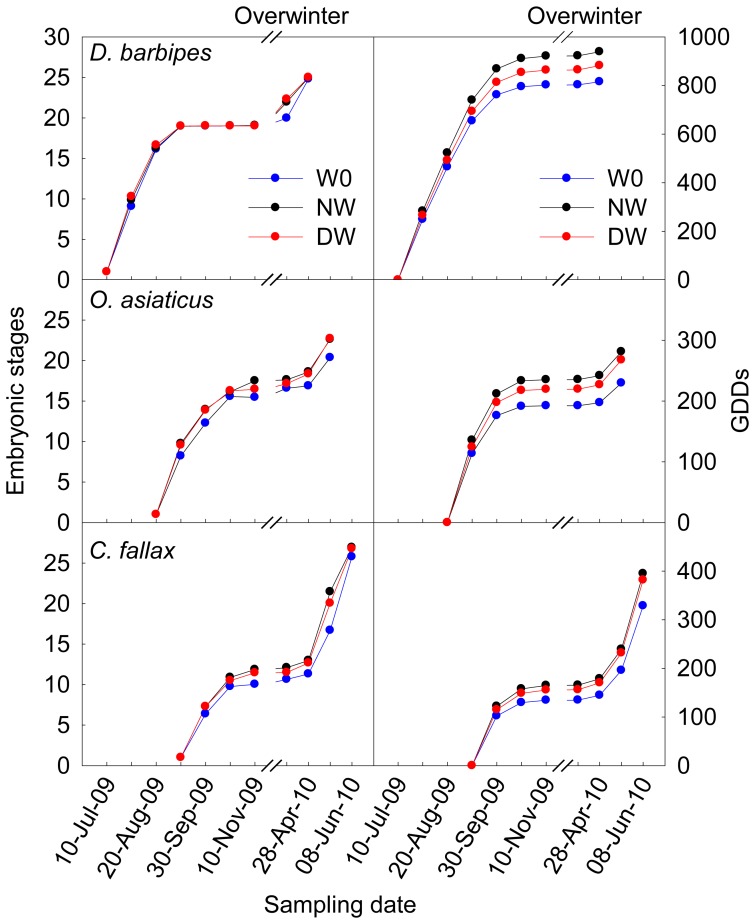
Embryonic development and accumulated growing degree days (GDDs) in *Dasyhippus barbipe*s, *Oedaleus asiaticus*, and *Chorthippus fallax* during the experiment. The arrow indicates the time of egg being embed into the soil. Note: W0, control; DW, daytime warming; NW, nighttime warming.

**Figure 4:**
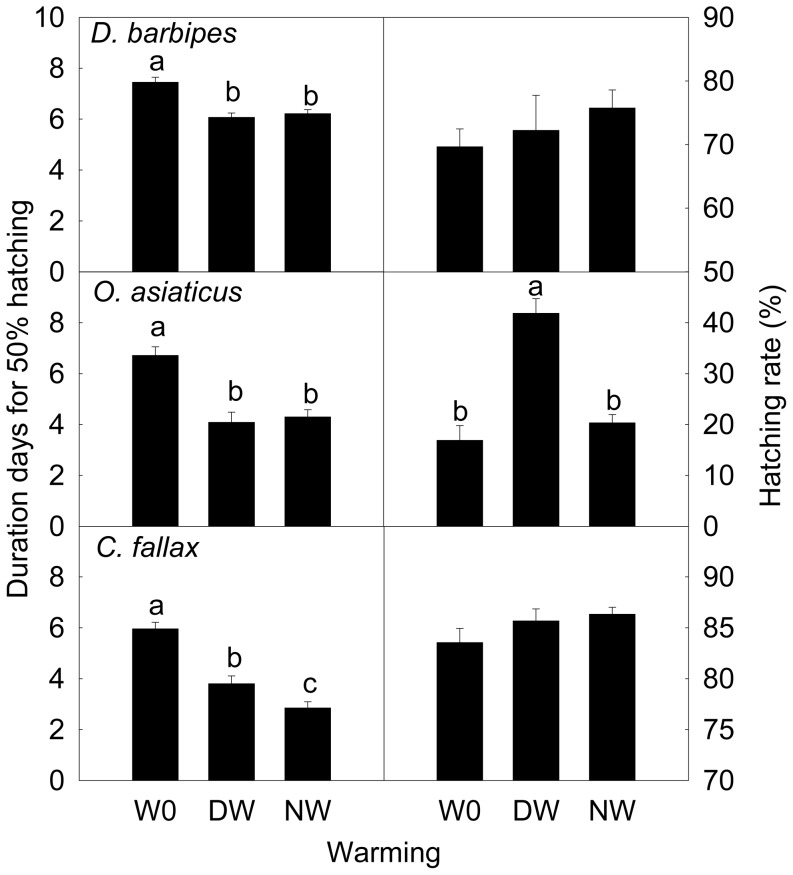
Duration for 50% hatching (left) and hatching rate (right) in *Dasyhippus barbipe*s, *Oedaleus asiaticus*, and *Chorthippus fallax*. Data are mean ± SE. Different letters indicate significantly different at *P*<0.05. Note: W0, control; DW, daytime warming; NW, nighttime warming.

Results of repeated measure ANOVA indicate that both daytime and nighttime warming advanced the embryonic development in the three grasshopper species, albeit varying patterns of the time course (*D. b*: *F* = 17.96, *P*<0.001; *O. a*: *F* = 20.46, *P*<0.001; *C. f*: *F* = 52.48, *P*<0.001, Fig. 3). In comparison with the treatments under ambient conditions, the average embryonic developmental stages was 2.90% and 2.41% more advanced under the daytime and nighttime warming, respectively, in *D. barbipes*, 8.57% and 10.3% more advanced in *O. asiaticus*, and 10.7% and 14.9% more advanced in *C. fallax*, over the entire embryonic developmental period. The effect of nighttime warming was greater than the daytime warming on the embryonic development in *C. fallax*; whereas the two warming regimes did not differ in their effects in *D. barbipes* and *O. asiaticus*. For all the three grasshopper species, warming markedly advanced the hatching time (*D. b*: *F* = 8.07, *P*<0.001; *O. a*: *F* = 19.87, *P* = 0.001; *C. f*: *F* = 33.61, *P* = 0.001, Fig. 4). Daytime warming advanced the hatching time by 1.38 days in *D. barbipes*, 2.63 days in *O. asiaticus*, and 2.40 days in *C. fallax*; whilst the nighttime warming advanced the hatching time by 1.23 days in *D. barbipes*, 2.42 days in *O. asiaticus*, and 3.11 days in *C. fallax*. Statistical analyses indicate that nighttime warming had a greater effect than the daytime warming on the hatching time in *C. fallax* (*P* = 0.031), but not in *D. barbipes* and *O. asiaticus* (Fig. 4).

The egg hatching rate in *O. asiaticus* (51.43%; *F* = 14.69, *P* = 0.002, Fig. 4) was significantly increased by 1.48 times by the daytime warming. No apparent effects of warming on egg hatching rate were detected in *D. barbipes* (*F* = 0.69, *P* = 0.55) and *C. fallax* (*F* = 1.60, *P* = 0.24) (Fig. 4).

### Growing degree days of egg development

In all the three species, the accumulated growing degree days (GDDs) during the period of embryonic development were increased as a result of warming treatments (*D. b*: *F* = 36.01, *P*<0.001; *O. a*: *F* = 26.10, *P* = 0.001; *C. f*: *F* = 26.74, *P* = 0.001, Fig. 3). Nighttime warming had a significantly greater effect on the total GDDs than the daytime warming in *D. barbipes*; whilst the total GDDs were not differentially affected by the daytime and nighttime warming regimes in *O. asiaticus* and *C. fallax*. Daytime warming increased the total GDDs by 8.7% in *D. barbipes*, 16.4% in *O. asiaticus*, and 20.1% in *C. fallax*; whereas nighttime warming increased the total GDDs by 15.2% in *D. barbipes*, 21.4%in *O. asiaticus*, and 16.1% in *C. fallax*.

We further analyzed the effects of warming on accumulated GDDs separately as pre-overwintering and post-overwintering. Daytime and nighttime warming significantly enhanced the GDDs of both pre-overwintering (*D. b*: *F* = 47.73, *P* = 0.0002; *O. a*: *F* = 30.42, *P* = 0.001; *C. f*: *F* = 21.27, *P* = 0.002, Fig. 3) and post-overwintering (*D. b*: *F* = 5.95, *P* = 0.04; *O. a*: *F* = 12.60, *P* = 0.007; *C. f*: *F* = 21.19, *P* = 0.002, Fig. 3) in the three grasshopper species. The warming effects on GDDs of post-overwintering did not differ between daytime and nighttime warming in the three species. Nighttime warming had a greater effect on elevating the pre-wintering GDDs than daytime warming in both *D. barbipes* and *O. asiaticus* (Fig. 3). In *D. barbipe*s, daytime and nighttime warming increased the pre-overwintering GDDs by 7.59% and 14.8% and that of post-overwintering by 23.2% and 20.1%, respectively. In *O. asiaticus*, daytime and nighttime warming increased the pre-overwintering GDDs by 22.3% and 14.1% and the post-overwintering GDDs by 19.2% and 22.2%, respectively. In *C. fallax*, daytime and nighttime warming increased the pre-overwintering GDDs by 22.6% and 15.7% and the post-overwintering GDDs by 18.4% and 16.5%, respectively (Fig. 3).

**Figure 5:**
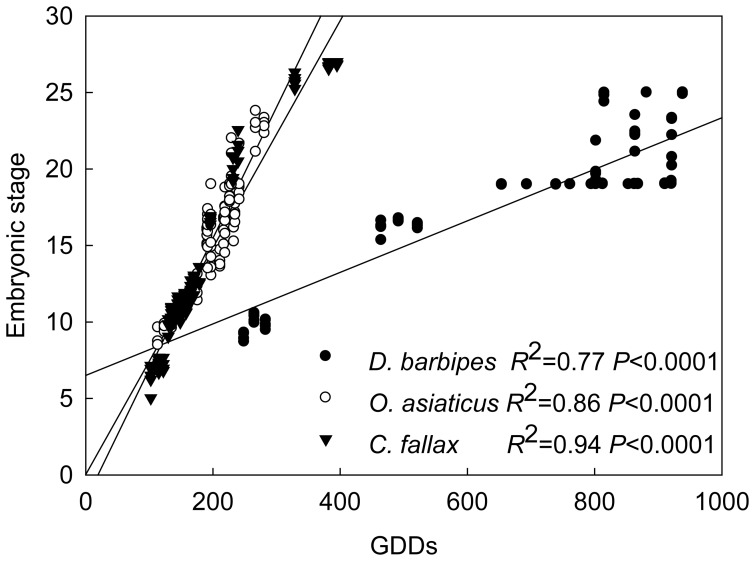
Relationships between embryonic stage and the total accumulative growth degree days (GDDs) during period of eggs development in *Dasyhippus barbipes*, *Oedaleus asiaticus*, and *Chorthippus fallax*.

Among the three grasshopper species, the embryonic development in the early-season *D. barbipes* had significantly lower (*P*<0.001) response (*R* ^2^ = 0.77, slope = 0.016) to GDDs than the mid-season *O. asiaticus* (*R* ^2^ = 0.86, slope = 0.086) and the late-season *C. fallax* (*R* ^2^ = 0.94, slope = 0.074); whilst the mid-season *O. asiaticus* had stronger (*P*<0.05) response to GDDs than the late-season *C. fallax* (Fig. 5).

### Nymph development and emergence rate

**Figure 6:**
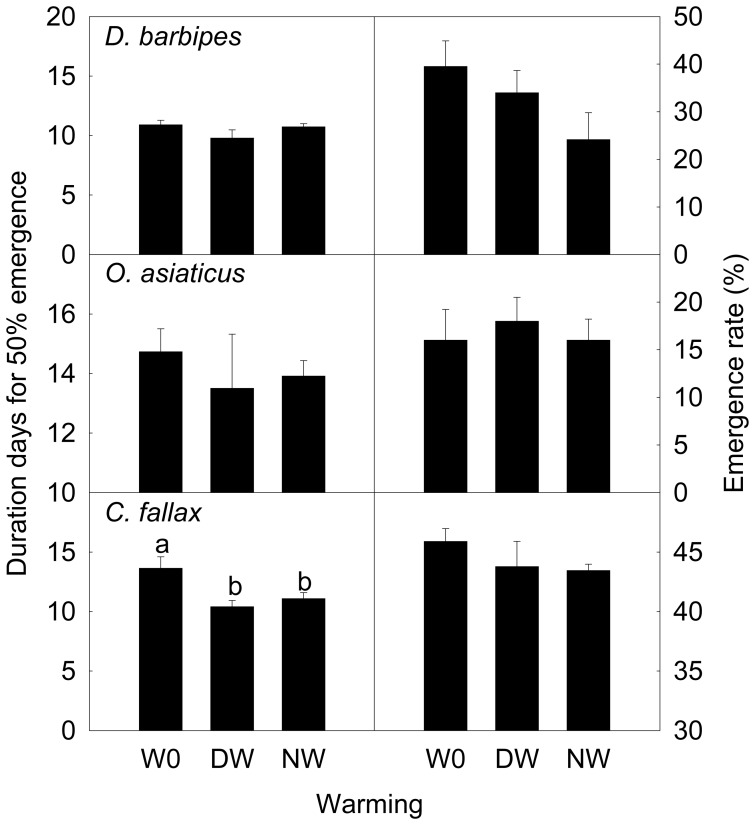
Duration for 50% emergence and emergence rate in *Dasyhippus barbipe*s, *Oedaleus asiaticus*, and *Chorthippus fallax* under different warming treatments. Data are mean ± SE. Different letters indicate significantly different at *P*<0.05. Note: W0, control; DW, daytime warming; NW, nighttime warming.

The nymph emergence time was significantly advanced by warming in *C. fallax* (*F* = 5.48, *P* = 0.02); it was advanced by 3.22 days by the daytime warming and 2.54 days by the nighttime warming. There was no significant difference between the two warming regimes in their effects on the emergence time (Fig. 6). Warming had no effect on nymph emergence time in *D. barbipes* (*F* = 1.60, *P* = 0.24) and *O. asiaticus* (*F* = 0.28, *P* = 0.76).

There was no significant effect of warming on the emergence rate in the three grasshopper species.

### Survival and fecundity of adults

**Figure 7:**
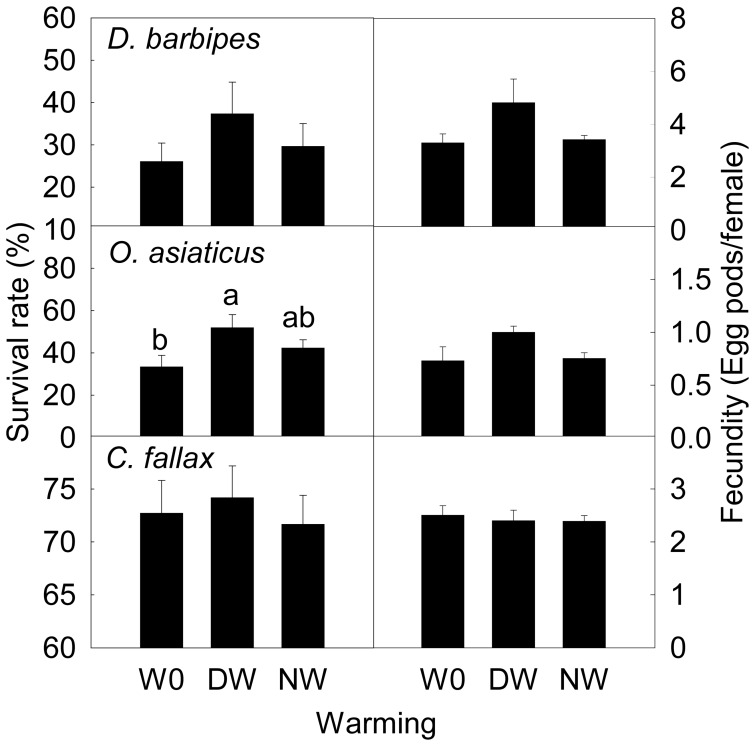
The adult survival rate and fecundity (number of egg pod per female) in *Dasyhippus barbipe*s, *Oedaleus asiaticus*, and *Chorthippus fallax* under different warming treatments. Data are mean ± SE. Different letters indicate significantly different at *P*<0.05. Note: W0, control; DW, daytime warming; NW, nighttime warming.

The warming treatments did not affect survival and fecundity of adults in the three grasshopper species except for adult survival rate in *O. asiaticus*, which was increased by 55% by daytime warming (*F* = 3.10, *P* = 0.08) (Fig. 7).

## Discussion

Both daytime and nighttime warming treatments were effective in raising soil and air temperatures in this study. However, a nighttime warming regime appeared to be more effective in increasing soil temperature than daytime warming, especially the minimum soil temperature; whereas a daytime warming regime was found to be more effective in increasing air temperature than nighttime warming. The temperature impacts of daytime vs. nighttime warming conform to a pattern of asymmetric change in diel temperature as previously identified through meteorological data analysis [3], [4], [24].

We found that warming in general advanced the developmental rate of eggs and nymphs, but had no apparent impacts on the hatching rate of eggs, emergence rate of nymph and survival and fecundity of adults in the three grasshopper species studied. Nighttime warming was more effective than the daytime warming in advancing egg development; whereas daytime warming was more effective than the nighttime warming in affecting nymph development.

In *C. fallax*, which is of non-diapause and late-season occurrence, nighttime warming had greater effect than daytime warming in advancing embryonic development and the timing of hatching and emergence. Such differential impacts of daytime vs. nighttime warming were not observed in the early-season *D. barbipes* and mid-season *O. asiaticus*, which are of obligatory and facultative diapause, respectively. Among the three species, the early-season *D. barbipes* was least affected by warming; whereas the effects did not much differ between the mid-season *O. asiaticus* and late-season *C. fallax* (Fig. 4). This may be because that the accumulated effects of warming were interrupted by diapause, leading to the advancement of hatching in *D. barbipes* mainly attributable to the thermal effects of warming during post-overwintering development. The post-overwintering GDDs in the early-season *D. barbipes* were only increased by 14.5 and 12.5 by the daytime and nighttime warming, respectively. Moreover, the early-season *D. barbipes* had a pre-overwintering GDDs of more than 800, but the eggs in this species entered into diapause at the embryonic stage 19, at which time the GDDs was about 500, meaning that approximately 300 GDDs was not effective in advancing the embryonic development in *D. barbipes*. These results suggest that diapause can limit the degree to which elevated temperature accelerates the embryonic development and acts as a protective mechanism of early-season grasshopper for avoiding the risk of pre-winter hatching, because there will be no sufficient thermal time permitting the completion of next generation [25]. This appears to be consistent with the differences in thermal sensitivity among the three grasshopper species in embryonic development as revealed by the relationship between embryonic stage and corresponding GDDs (Fig. 5).

Results in this study provide a clear evidence that the responses of the three grasshopper species to warming are affected by several factors, including species traits, developing stage, and the thermal sensitivity, but not simply a thermal effect, contrary to the suggestion by Nufio *et al.* (2010) that phenological advancement in grasshoppers would depend on when a set number of GDDs is reached during a season [26]. Here with a field climate manipulation experiment, we found that advancements in the embryonic development occurred along with markedly increased GDDs only for non-diapause grasshopper. Apparently, there is a more complex mechanism to drive embryonic development than just a thermal effect in grasshoppers. The varied responses to warming and contrasting warming regimes among different life stages may attribute to differences in living environments. Generally, the developmental rate of insects is temperature dependent within a suitable temperature range [27]–[29]. Grasshopper eggs stay in soil during development, and are stationary and passive to receive the environmental changes [30], but nymphs and adults can move around in response to changing environmental conditions, do not experience wide range of temperature by thermoregulation. Hence there are greater responses to variable warming in eggs than during other life stages. Moreover, as the nighttime and daytime warming regimes result in significantly distinct microclimate changes in soil and air, an asymmetric warming would impose differential impacts on the development of eggs and hoppers.

The seasonal timing of warming could also impose differential impacts on various organisms, which may explain in part the contrasting patterns of responses among the three grasshopper species in this study. It has been found that in the lake plankton species, the life history strategies and seasonal pattern of warming together determine the responses to climate change [27], [31], [32]. By examining the hourly soil and air temperature variations during the experimental period, we found that the effectiveness of the warming treatments varies seasonally. The temperatures in our study are all below the lower developmental threshold (i.e. ≤10°C for the grasshoppers in this study) in winter, and may occasionally rise above the upper developmental threshold (i.e. 40°C) in summer (Fig. S1). The Inner Mongolian grasslands are in the temperate climatic zone and have mostly cool nights, with nighttime temperatures at around 20°C even in the hottest summer days (Fig. S2). It is therefore reasonable to expect a great impact of the nighttime warming on the local grasshoppers than that of the daytime warming. At the early or late growth season, both the daytime and the nighttime warming are effective to facilitate the temperature requirements of grasshoppers; whereas the impact of daytime warming in summer can be none or negligible because of the already high temperatures.

Research to date shows varied phenological responses to warming in insects. While many species display phenological advancement in response to warming [33]–[38], there are also species not much affected by warming or reacting with delayed phenology [17], [26], [39]–[41]. There are findings of either greater responses in species of spring occurrence than species that occur later in the season [34], [42]–[45] or an opposite pattern [17], [26]. In this study, we found that the adult occurring time (advance in egg hatching+advance in nymph emergence) of the early-season *D. barbipes* was not significantly advanced by warming treatment (2.5 days by daytime warming and 1.5 days by nighttime warming), and that of mid-season *O. asiaticus* and the late-season *C. fallax* was significantly advanced (3.86 days and 5.62 day by daytime warming, and 3.23 days and 5.65 days by nighttime warming, respectively). This phenological advancement was associated with increasing GDDs.

The responses in grasshopper development to warming appear to differ with that of plants. Warming advanced the flowering time in early- and mid-blooming plant species, not in the late-blooming plant species [46]. However, in our study, the mid- and late-season grasshopper species were more susceptible than the early-season species to warming. Our results with grasshoppers showed greater phenological responses to warming than reported for flowering plants [46]. Such mismatch in the phenological responses to warming between insects and their host plants can have detrimental impacts on ecosystem functioning.

In our experiment, the temperature was only increased by less than 0.5°C in the air (0.23°C by daytime warming and 0.34°C by nighttime warming) and by 1°C in soils (0.59°C by daytime warming and 1.14°C by nighttime warming in soils). With such small increases in temperature, the phenology in the grasshopper species advanced by more than 2–5 days. If the predicted temperature increase of 6.4°C at the end of this century occur [47], the impact on grasshoppers could be much greater. The species interaction relationship could be disrupted, leading to changes in the grassland community structure and functioning [46].

Overall, findings in this study highlight the importance of life-history strategies in determining specific responses to warming, and indicate complex relationships between climate change and responses in invertebrate. For prediction of ecosystem response to climate change, it is important to consider the differential impacts of daytime and nighttime warming on phenology in different life forms.

## Materials and Methods

### Ethics Statement

No specific permits were required for the described field studies. The all field experiments were conducted in Duolun Restoration Ecology Experimentation and Demonstration Station of the Institute of Botany, Chinese Academy of Sciences. The location is not privately-owned or protected in any way, and the field studies did not involve endangered or protected species.

### Study site

The research was conducted at a field site (42°02′N, 116°17′E, 1324 m a.s.l) in Duolun County, Inner Mongolia. The long-term (1953–2007) mean annual temperature of the study area is 2.1°C and monthly mean temperature ranges from 18.9°C in July to −17.5°C in January. The annual mean of daily temperature range (DTR, defined as a difference between the mean daily maximum temperature and the mean daily minimum temperature) is 13.7°C, varying from 12.1°C in December to 15.5°C in April. Mean annual precipitation is 383 mm, of which 90% occur between May and October. Soil is of sandy texture and classified as Haplic Calcisols according to the FAO classification. Mean soil bulk density is 1.31 g cm^−3^ and pH is 6.84±0.07.

### Test grasshoppers

Three grasshopper species of different life-history traits were used in this study, including an early-season species *Dasyhippus barbipes* (F.-W), a mid-season species *Oedaleus asiaticus* B.-bienko, and a late-season species *Chorthippus fallax* (Zub). *Dasyhippus barbipes* is of obligatory diapause, with eggs hatching in early May and adult population peaking in mid-June; overwinter eggs all enter diapause at embryonic stage 19 in late autumn. *Oedaleus asiaticus* is of facultative diapause, with eggs hatching in early June and adult population peaking in mid-July; only partial overwintering eggs enter diapause at stage 19, with others continuing their development to complete blastokinesis if temperature permits. *Chorthippus fallax* is of non-diapause, overwintering at earlier embryonic stages (stage 3) [48], [49].

### Experimental setup and warming treatments

The field experiment was set up with a completely randomized arrangement, with treatments consisting ambient temperature condition (W0), daytime warming (DW: 06:00–18:00 h Beijing Time), and nighttime warming (NW: 18:00–06:00 h), each with five replicated 3 m×4 m plots. Warming on each plot was achieved with a 165 cm×15 cm MSR-2420 infrared heater (Kalglo Electronics Inc, Bethlehem, PA, USA) suspended 1.85 m above the ground. A “dummy heater” with the same shape and size as the infrared heater was used to control for the shading effect. The warming treatments were carried out from July 1 through November 11 in 2009 and from March 13 through June 7 in 2010.

### Processing of test materials

In 2008, the adults of the three grasshopper species were collected during their respective peak occurrences, and reared in cages for producing egg pods under controlled laboratory conditions. The rearing cages were maintained at 30±1°C during the daytime and 25±1°C at night, with a day/night regime of 14/10 h and sand placed on the bottom as oviposition substrate. The egg pods were collected daily and placed in a refrigerator at 5°C until being deployed in the field warming experiment.

Prior to the field experiment, we thoroughly mixed all egg pods collected at different times to eliminate the effect of oviposition date on hatching in the following year [50], and placed 20 egg pods in each paper cup filled with moist sand. The egg pods were embedded underneath a 3–5 cm layer of sand in paper cups; 10 paper cups with egg pods for each species were buried in soils of every treatment plots with the top leveling with the ground surface.

### Soil temperature and moisture

During the field experiment, soil temperature and moisture at a depth of 5 cm, and air temperature and humidity at 10 cm above the ground, were automatically monitored within each plot and data recorded with a data logger (CR1000, Campbell Science Equipment, Logan, UT, USA). Soil temperature and volumetric water content (V/V %) were measured respectively using thermocouples and segmented CS616 (Campbell Science Equipment, Logan, UT, USA). All measurements were made at 10-minute intervals and data recorded as the maximum and minimum values as well as the hourly means.

### Egg development and hatching

The embryonic development was examined at 20-day intervals following commencement of the field experiment until eggs entered into overwintering in 2009 and from April 7, 2010 until the eggs were about to hatch. At each sampling time, one set of egg samples (*i.e.* in one paper cup) were retrieved from each treatment plot by species and dissected under a microscope for determination of the embryonic stage. If an egg turned flaccid, brown, or moldy, it was considered dead; whereas the cream-colored and turgid eggs were considered alive [23]. The overall embryonic development was divided into 27 stages according to the morphological traits of each stage [51].

When majority of the eggs reached embryonic stage 25–26, all egg samples were transferred from the field into incubators in laboratory and cultivated at 26°C for 60 days. The hatchlings were counted daily until after 60 days when all the egg pods were shelled off. The living and dead eggs were then classified and counted. In this study, we refer hatching time as the time required for observing 50% egg hatching.

### Growing degree days (GDDs) for egg development

GDDs are a measure of the physiological time that is required for an insect to complete a specific developmental stage. They are defined as accumulated daily heat units in a temperature range above a lower threshold for commencing essential metabolic processes but below an upper threshold for cessation of biological activities in a given organism. We calculated the GDDs for egg development by subtracting the lower threshold temperature for each grasshopper species from the hourly mean soil temperature by treatment plots. The lower threshold temperatures (LDT) were previously determined as 9.5°C in *D. barbipes*, 11.5°C in *O. asiaticus* and 10.5°C in *C. fallax* [22], [23]. The upper threshold temperature is estimated at 40°C for these grasshopper species.

### Nymph development

Eggs were collected from the adult rearing cages and placed in outdoor soils for overwintering in 2007. In the following year, those eggs were transferred into incubators at the beginning of growth season and maintained at 30°C until natural hatching. First instars' nymphs were then collected and transferred into field cages on each treatment plot within two days. Each cage contained 100 individual nymphs in *D. barbipes* and *C. fallax* and 50 in *O. asiaticus*. Nymphs in the field cages were fed with fresh wheat seedlings daily and their developmental stages were examined at 5-day intervals. The molted adults were counted and removed daily until all nymphs completed their emergence. We determined the nymph development time as the time required for observing 50% adults.

### Survival and fecundity of adults

During the emergence period, 70 adults in *D. barbipes* and *C. fallax* and 40 in *O. asiaticus* (♀∶ ♂ = 1∶1) were transferred into a separate case on each treatment plot. After one month, the surviving individuals were counted and removed out of the cages. Egg pods were collected from each cage and examined for fecundity (the average number of egg pods per female).

## Data Analysis

One way analysis of variance (ANOVA) was used to assess the effects of daytime vs. nighttime warming on the soil temperature and moisture, the hatching time and rate of the eggs, the total accumulated GDDs, pre-overwintering GDDs and post-overwintering GDDs during embryonic development period, the developmental time and emergence rate of nymph, survival rate and fecundity of the adults. In this study, the timing for the first observed hatching and emergence was used as the first day in the analysis of duration for 50% hatching and emergence. The repeated measures ANOVA was used to examine the effects of daytime and nighttime warming on the embryonic development. We compared the thermal sensitivity among the three grasshopper species and between different warming treatments in embryonic development by performing linear regression analysis of embryonic stage vs. corresponding GDDs. All statistical analyses were made with SPSS16.0 (SPSS Inc., USA) and the differences between treatments are considered significant if *P*≤0.05.

## Supporting Information

Figure S1 **Data of daily mean, maximum and minimum temperatures at 5 cm belowground and 10 cm aboveground in control plots during experiment.** The dashed lines indicate the lower, optimum and upper temperature limits for grasshopper development.
(TIF)

Figure S2 **Data of the mean soil temperatures at 5 cm belowground of daytime and nighttime in control plots during experiment.**
(TIF)
