# Supplementary figures and images for: Specificity Responses of Grasshoppers in Temperate Grasslands to Diel Asymmetric Warming
Source: PLoS One. 2012 Jul 27;7(7):e41764. doi: 10.1371/journal.pone.0041764 (PMC3407111; doi:10.1371/journal.pone.0041764)

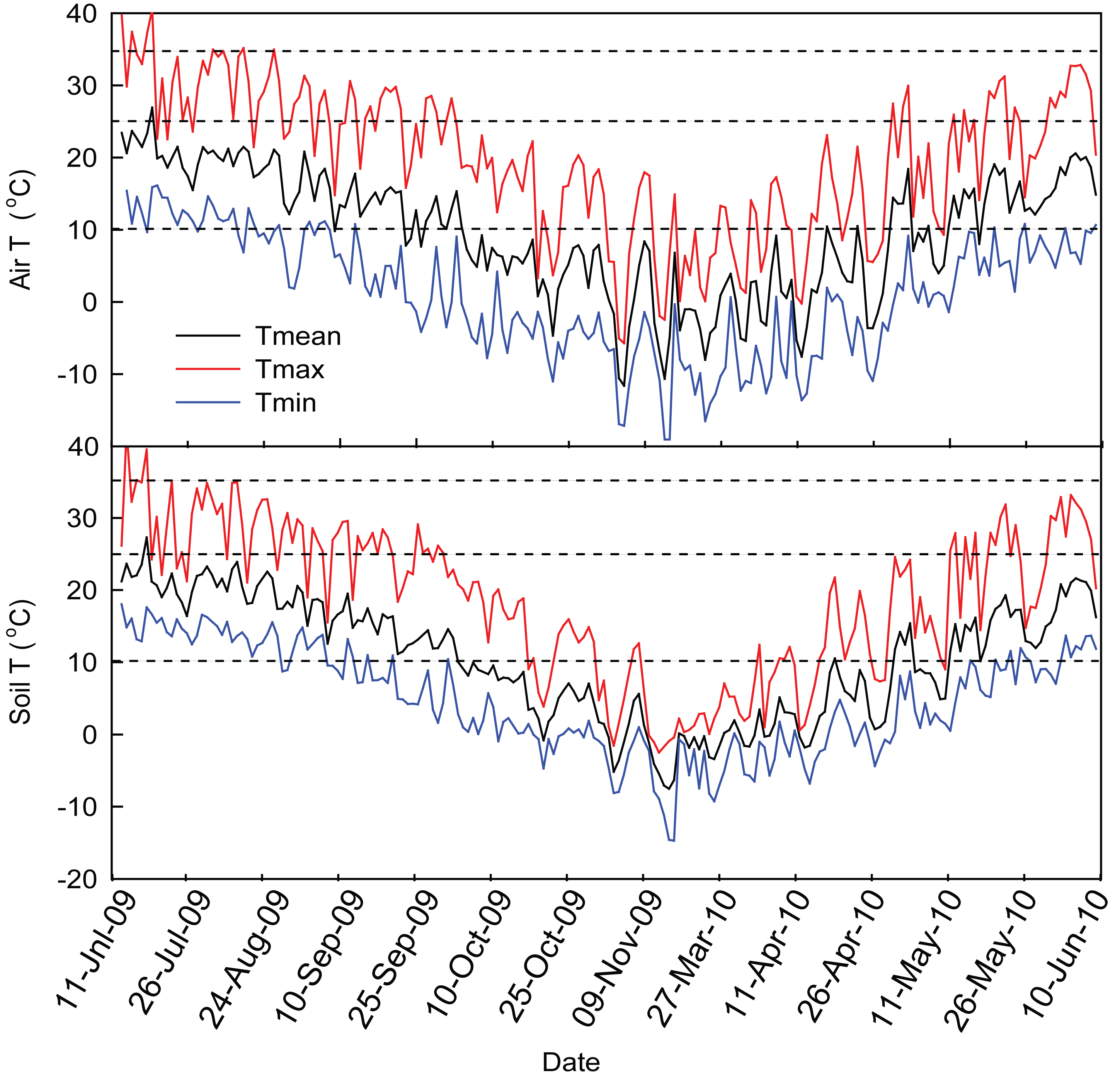

Supplement: Figure S1 — Data of daily mean, maximum and minimum temperatures at 5 cm belowground and 10 cm aboveground in control plots during experiment. The dashed lines indicate the lower, optimum and upper temperature limits for grasshopper development. (TIF) [file pone.0041764.s001.tif]

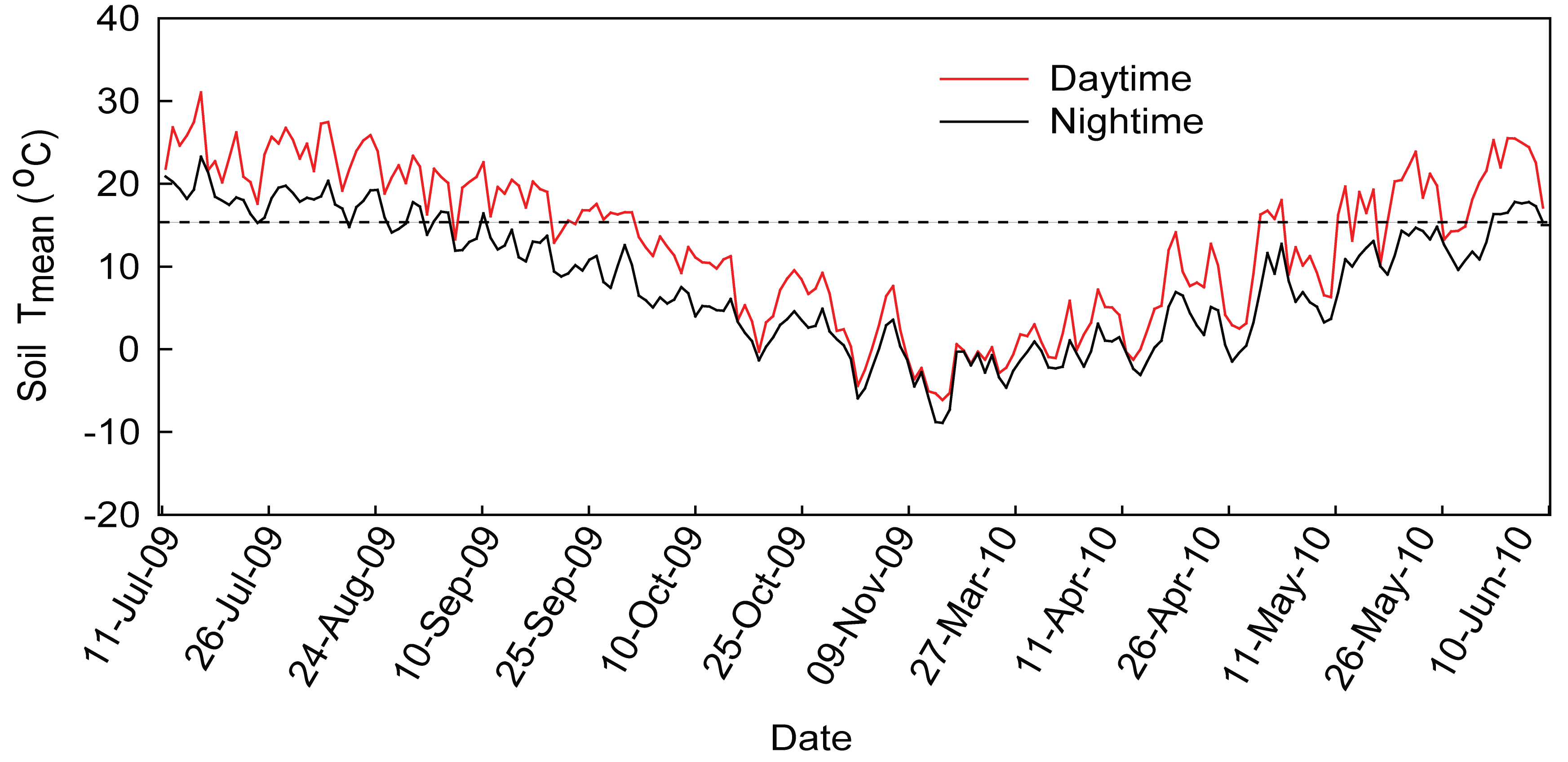

Supplement: Figure S2 — Data of the mean soil temperatures at 5 cm belowground of daytime and nighttime in control plots during experiment. (TIF) [file pone.0041764.s002.tif]
